# Malaria prevalence and long-lasting insecticidal net use in rural western Uganda: results of a cross-sectional survey conducted in an area of highly variable malaria transmission intensity

**DOI:** 10.1186/s12936-021-03835-7

**Published:** 2021-07-05

**Authors:** Claire M. Cote, Varun Goel, Rabbison Muhindo, Emmanuel Baguma, Moses Ntaro, Bonnie E. Shook-Sa, Raquel Reyes, Sarah G. Staedke, Edgar M. Mulogo, Ross M. Boyce

**Affiliations:** 1 Gillings School of Global Public Health, University of North Carolina at Chapel Hill, Chapel Hill, NC USA; 2 Department of Geography, University of North Carolina at Chapel Hill, Chapel Hill, NC USA; 3 Carolina Population Center, University of North Carolina at Chapel Hill, Chapel Hill, NC USA; 4 Department of Community Health, Mbarara University of Science and Technology, Mbarara, Uganda; 5 Department of Biostatistics, Gillings School of Global Public Health, University of North Carolina at Chapel Hill, Chapel Hill, NC USA; 6 Division of Hospital Medicine, UNC School of Medicine, University of North Carolina at Chapel Hill, Chapel Hill, NC USA; 7 London School of Hygiene and Tropical Medicine, London, UK; 8 Institute of Global Health and Infectious Diseases, UNC School of Medicine, University of North Carolina at Chapel Hill, 130 Mason Farm Road, CB 7030, Chapel Hill, NC 27599 USA

**Keywords:** Malaria, Plasmodium, Mosquito nets, Insecticide-treated bed nets, Uganda

## Abstract

**Background:**

Long-lasting insecticidal nets (LLINs) remain a cornerstone of malaria control, but strategies to sustain universal coverage and high rates of use are not well-defined. A more complete understanding of context-specific factors, including transmission intensity and access to health facilities, may inform sub-district distribution approaches and tailored messaging campaigns.

**Methods:**

A cross-sectional survey of 2190 households was conducted in a single sub-county of western Uganda that experiences highly variable malaria transmission intensity. The survey was carried out approximately 3 years after the most recent mass distribution campaign. At each household, study staff documented reported LLIN use and source among children 2 to 10 years of age and performed a malaria rapid diagnostic test. Elevation and distance to the nearest health facility was estimated for each household. Associations between parasite prevalence and LLIN use were estimated from log binomial regression models with elevation and distance to clinic being the primary variables of interest.

**Results:**

Overall, 6.8% (148 of 2170) of children age 2–10 years of age had a positive RDT result, yielding a weighted estimate of 5.8% (95% confidence interval [CI] 5.4–6.2%). There was substantial variability in the positivity rates among villages, with the highest elevation villages having lower prevalence than lowest-elevation villages (*p* < .001). Only 64.7% (95% CI 64.0–65.5%) of children were reported to have slept under a LLIN the previous night. Compared to those living < 1 km from a health centre, households at ≥ 2 km were less likely to report the child sleeping under a LLIN (RR 0.86, 95% CI 0.83–0.89, *p* < .001). Households located farther from a health centre received a higher proportion of LLINs from government distributions compared to households living closer to health centres.

**Conclusions:**

LLIN use and sourcing was correlated with household elevation and estimated distance to the nearest health facility. The findings suggest that current facility-based distribution strategies are limited in their reach. More frequent mass distribution campaigns and complementary approaches are likely required to maintain universal LLIN coverage and high rates of use among children in rural Uganda.

**Supplementary Information:**

The online version contains supplementary material available at 10.1186/s12936-021-03835-7.

## Background

Malaria remains an important cause of global morbidity and mortality despite substantial gains against the disease over the past two decades [1]. Much of the progress against malaria can be attributed to the development and widespread implementation of long-lasting insecticidal nets (LLINs) [2]. When widely distributed in the community and used in the household, LLINs provide both a physical barrier against the bite of female *Anopheles* mosquitoes as well as a killing effect (i.e., vector control) resulting from contact between the mosquito and the impregnated insecticide [3]. Yet the emergence of resistance to pyrethroid insecticides, including permethrin and deltamethrin, threatens many of these gains [4]. Recent reports suggest that global progress against malaria has stalled and may even be slipping backwards among high-burden countries in sub-Saharan Africa (SSA) [5]. Nets employing novel insecticides or combinations of insecticides have shown to be effective in settings with established insecticide resistance, but these are not yet widely deployed [6, 7]. Therefore, continued focus on the development of effective implementation strategies to achieve universal coverage, which the World Health Organization (WHO) defines as one LLIN for every two persons at risk of malaria, and high rates of use remains a critical undertaking [8].

Among malaria-endemic countries in SSA, Uganda has been a leader in the effort to achieve universal coverage [9]. Uganda conducted its first mass distribution campaign in 2013, with over 20 million LLINs distributed [10]. This effort was followed by similar campaigns every 3 years, including in 2017–18 and most recently in 2020–21 in accordance with WHO guidelines [8]. Households reporting at least one LLIN increased from 16% in the 2006 Demographic and Health Survey (DHS) to more than 80% in the 2018 Malaria Indicator Survey, while over the same period the proportion of households with at least one LLIN for every two people increased from 5 to 54% [11]. Furthermore, in the years immediately following the initial distribution campaign, substantial reductions in malaria parasite prevalence and disease burden were observed [12]. Towards the end of each 3-year cycle, however, attrition due to physical damage and degradation—which begins as soon as the LLINs leave the factory—can leave households well below universal coverage targets with a resulting increase in malaria transmission intensity [13, 14].

To maintain coverage between mass distribution campaigns, the WHO recommends continuous LLIN distribution through antenatal care clinics and the expanded programme on immunization. These channels, which leverage public health services utilized by at-risk populations (e.g., pregnant women and young children), aim to fill coverage gaps that emerge due to changes in the population due to births and migration in the interval period between mass distribution campaigns. However, strategies to replace LLINs that experience premature attrition are not as well-defined. This may be partly attributable to the high cost of monitoring LLIN durability and performing gap analysis [15, 16]. At present, the WHO does not recommend replacement or “top-up” campaigns because “accurate quantification for such campaigns is generally not feasible and the cost of accounting for existing nets outweighs the benefits [8].”

A much smaller proportion of the existing literature has examined the effectiveness of LLIN distribution outside of mass distribution campaigns, [17–22] particularly in regard to geographic factors that may impact the coverage and use. While analysis of routine DHS data from 25 countries found that facility-based distribution improves LLIN ownership rates and reported use [23], a study in rural Kenya found that increased distance from health facilities was associated with decreased LLIN ownership [24] and another a study in Malawi found that households further from health facilities were less likely to own a LLIN and have their child sleep under it [25] Therefore, as part of an ongoing, cross-sectional study of malaria transmission in the western Ugandan highlands, the study team sought to examine how geographic factors, including elevation and distance to clinic, might influence malaria risk and LLIN sourcing and use. While these factors may represent surrogate measures of transmission intensity and facility utilization, respectively, the relative accessibility of such data may be particularly useful to inform implementation strategies.

## Methods

### Study site

**Fig. 1:**
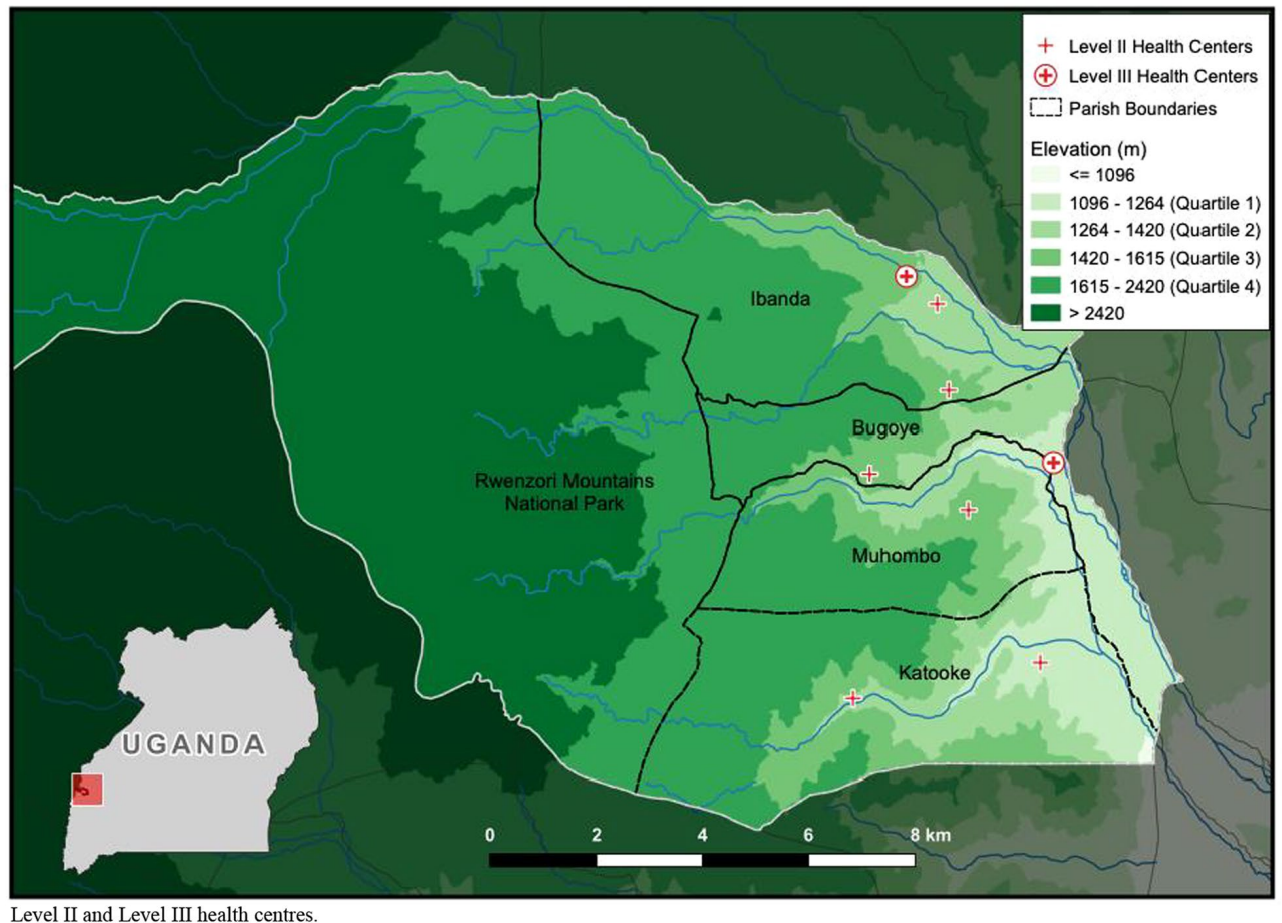
Elevation map of Bugoye sub-county displaying parish boundaries and location of Level II and Level III health centres

The Bugoye sub-county, located in the Kasese District of Western Uganda is comprised of 35 villages, spanning a rural, highland area of approximately 55 km^2^. The population of the sub-county is 50,249, approximately one-quarter of whom are children under 5 years of age [16]. The geography of the sub-county is highly varied and characterized by deep river valleys and steep hillsides with elevations up to 2500 m (Fig. 1).

The sub-county’s primary public health facility is the Bugoye Level III Health Centre (BHC). BHC is comprised of a 25-bed inpatient ward, where patients can receive intravenous medications, a busy outpatient clinic that evaluates 60–80 patients per day, a maternity ward, and a small laboratory capable of performing point-of-care tests for diseases such as malaria and HIV. There are also level II health centres in each of the six parishes that offer basic outpatient services including routine vaccination, and one private-not-for-profit level III health centre operated by the Rwenzori Mountaineering Services.

The climate in Bugoye permits year-round malaria transmission marked by semi-annual transmission peaks typically following the end of the rainy seasons in May and December [26]. The most recent malaria indicator surveys (MIS) undertaken in the Mid-Western region (2014–15) and Tooro sub-national region (2018–19) which include Bugoye, reported *Plasmodium falciparum* parasitaemia rates (PfPR) of 17.4% and 7.3%, respectively [11, 27]. The most recent mass distribution of LLINs took place in 2017 and is supplemented by ongoing distributions through antenatal and immunization clinics.

### Household survey

The study utilized a stratified random sample design with the village being the unit of stratification. Surveys were conducted in all 35 villages of Bugoye sub-county. Prior to each survey, community health workers (CHWs), each of whom is responsible for approximately 30–40 households, disseminated information about the aims and methods of the study to the residents of their respective coverage areas in an attempt to maximize participation. Starting from their own home, CHWs guided study staff in a clockwise direction to the nearest household with an eligible child (age 2–10 years). If multiple eligible children were present in the household, a random number generator was used to create an integer sequence with values between 2 and 10. The first child to have an age matching a number in the sequence was selected for testing. The CHW and staff then continued the survey in a systematic manner, stopping at every other house. If there was no adult present at the time of the visit, the survey team moved to the next eligible household.

After consent was provided, the study team administered a brief questionnaire that elicited responses about care-seeking behaviours, LLIN use and sourcing, and recent health (available in Additional file 1). Axillary temperature was measured in all children and 50 μl of capillary blood drawn for a malaria rapid diagnostic test (RDT) (SD Bioline Malaria Ag P.f., Abbott Laboratories, Chicago, IL, USA). The RDT is a qualitative test for the detection of histidine-rich protein II (HRP-II) antigen of *P. falciparum* in human whole blood [28]. RDT results were recorded as either positive or negative, with faint lines being considered positive. Results were provided to the consenting caregiver and recorded in the questionnaire.

All children with a history of fever in the prior 48 h or documented fever (axillary temperature ≥ 37.5 °C) at the time of initial evaluation and a positive RDT test result received weight-based treatment with artemether–lumefantrine [29]. Asymptomatic children with a positive RDT test result were not treated, which is consistent with current national guidelines [30]. Children with fever and a negative RDT test result were referred to the nearest public health facility for further evaluation.

### Data management & analysis

The sample size was estimated to achieve a coefficient of variation of approximately 20% for village-level malaria prevalence estimates. Based on these calculations, 60 eligible households in each village were surveyed—further stratified into twelve households per CHW in order to achieve spatial distribution within each village. CHWs and study staff first visited the nearest house to the CHW’s home, then moved in a clockwise direction, visiting every other household until the required number of households had been surveyed. All information was recorded in and uploaded to a secure electronic database (i.e. REDCap) using a portable tablet device [31]. Data were analysed using Stata version 16 (College Station, Texas). After the survey was complete, data was cleaned by manual review. Minor typographical errors were corrected for temperature, latitude, and longitude. Entries without evaluable latitude and longitude were excluded from further analysis.

The following outcome measures were assessed: (i) parasite prevalence or PfPR defined as the proportion of children with a positive malaria RDT result among all tests performed (ii) LLIN use among children, measured by asking the caregiver if the participating child slept under a LLIN the previous night, and (iii) the source of the LLIN. Weighted estimates of parasite prevalence and LLIN use were generated using the svyset command in Stata, which accounted for the estimated probability of selection for each household, sample stratification, and the finite population correction (FPC) factors [32]. Village population estimates were obtained from the most recent CHW census and were used to determine sampling weights and FPC factors. Unless stated otherwise, all estimates are weighted to the sub-county population. Weighted categorical outcomes were analyzed using Pearson’s Chi-squared test and binary outcomes were modeled using log-binomial regression to estimate crude and adjusted risk ratios (RR). A Wald test was used to assess the parameters of potential explanatory variables and those with p-values > 0.05 were not included in the adjusted models.

Elevation data for each household location was derived using the Google Elevation Application Programming Interface. Elevation quartiles were generated in Stata using the xtile command. Euclidean distances were calculated for both distance to nearest health centre (level II or III) and distance to nearest level III health centre. Distances were categorized by < 1 km, 1–2 km, and > 2 km to nearest health centre level II or III. The association between LLIN use and distance to health centres was estimated from a design-consistent log binomial regression model.

### Ethical approvals

Ethical approval of the study was provided by the institutional review boards of the University of North Carolina at Chapel Hill (19-1094), the Mbarara University of Science and Technology (06/03-19), and the Uganda National Council for Science and Technology (HS 2628). Adult caregivers provided written consent to participate in the study. Children ≥ 8 years of age were asked to provide written assent to participate.

## Results

From January 8 to March 11, 2020, field staff surveyed a total of 2190 households, representing 31.8% of all households in the sub-county. After removal of erroneous values, 99.2% (2173 of 2190) of entries had evaluable GPS coordinates, while malaria rapid diagnostic test results were available for 99.9% (2170 of 2173) of entries. Overall, 6.8% (148 of 2170) of children age 2–10 years of age had a positive RDT result, yielding a weighted estimate of 5.8% (95% confidence interval [CI] 5.4–6.2%). Yet, there was substantial variability in the positivity rates among villages, ranging from 0% (0 of 360) in six villages to a high of 31.7% (19 of 60) in Kansanzi village. A summary of household characteristics and malaria positivity prevalence (e.g., PfPR) stratified by elevation quartile is shown in Table 1. High-elevation villages had a lower PfPR than lower-elevation villages, and a smaller proportion of children with a self-reported fever had a positive RDT at the time of the survey.

**Table 1:** Summary of household characteristics stratified by elevation quartile

	Quartile 1	Quartile 2	Quartile 3	Quartile 4	*p*-value^*^
General					
Households (n)	544	543	543	543	–
Elevation range (m)	1096–1263	1264–1419	1420–1614	1615–2420	–
Median elevation (m, IQR)	1219 (1186–1241)	1362 (1318–1388)	1500 (1452–1555)	1737 (1674–1829)	–
Distance to health centre					
Less than 1 km	54.9 (53.7–56.1)	58.4 (57.2–59.7)	56.4 (55.3–57.6)	16.3 (15.3–17.3)	< .001
1 km to 2 km	37.0 (35.7–38.3)	41.2 (39.9–42.5)	33.3 (32.1–34.6)	38.1 (36.9–39.4)	
More than 2 km	8.1 (7.2–9.0)	0.4 (0.2–0.6)	10.2 (9.5–11.0)	45.6 (44.4–46.8)	
Care-seeking					
Sought care in past two weeks?	16.2 (15.0–17.5)	16.4 (15.3–17.5)	12.0 (11.1–13.0)	9.1 (8.2–10.1)	< .001
Location where care provided (n, %)					
Hospital	1 (1.1)	2 (2.4)	1 (1.5)	2 (4.1)	0.74
Health centre	47 (53.4)	42 (50.0)	36 (53.7)	21 (42.9)	
Pharmacy or drug shop	11 (12.5)	12 (14.3)	14 (20.9)	9 (18.4)	
Community health worker	26 (29.6)	27 (32.1)	15 (22.4)	17 (34.7)	
Traditional healer	3 (3.4)	1 (1.2)	1 (1.5)	0 (0.0)	
LLINs					
Child slept under LLIN last night?	65.6 (64.0–67.2)	69.8(68.4–71.2)	66.0 (64.6–67.4)	56.4(54.8–67.4)	< .001
LLIN source					
Government distribution	77.8 (76.1–79.5)	81.5 (80.1–82.9)	81.8 (80.4–83.1)	89.2 (87.8–90.4)	< .001
Health centre	22.2 (20.5–23.9)	16.9 (15.6–18.3)	18.2 (16.9–19.6	10.8 (9.6–12.2)	
Store/private vendor	–	1.6 (1.2–2.1)	–	–	
Fever and malaria					
Subjective fever in past two days?	9.3 (8.4–10.3)	10.0 (9.1–10.9)	5.3 (4.7–6.0)	5.2 (4.5–6.0)	< .001
PfPR	9.24 (8.35–10.22)	9.28 (8.42–10.20)	2.98 (2.52–3.52)	1.82 (1.42–2.32)	< .001

Unless otherwise indicated, data presented represents weighted proportion of households with corresponding 95% confidence intervals

* p-value from Pearson’s chi squared test for difference in proportions across elevation quartiles

Of those surveyed, 64.7% (95% CI 64.0–65.5%) of caregivers reported that the participating child slept under a LLIN the previous night. The vast majority of respondents reported obtaining the LLIN from either a distribution campaign (n = 1119, 82.1%) or a health facility (n = 265, 17.2%). Only four households reported purchasing a LLIN from a vendor. The proportion of children sleeping under a LLIN was similar in the sites of lowest elevation (Quartile 1 and Quartile 2, Table 1), but was lower in households at higher elevation when compared to the lowest quartiles.

Among households reporting LLIN use, an estimated 5.4% (95% CI 5.0–5.8%) had a positive RDT result, whereas 6.6% (95% CI 6.0–7.3) of children who were not reported to have slept under a LLIN had a positive RDT result *(p* = 0.002). In the unadjusted analysis, children who reported using LLINs were less likely to have a positive RDT result compared to children who did not use LLIN (RR 0.83, 95% CI 0.72–0.93). At lower elevation, higher transmission sites, the risk of a positive RDT result was greater in children who did not use LLIN compared to those who did. However, at the highest elevation sites the risk of a positive RDT was lower overall, but no difference in malaria risk was observed for children who used nets versus those who did not (Table 2).

**Table 2:** Results from unadjusted (left columns) and adjusted (right columns) log binomial regression modeling of a positive malaria RDT result

Variable	RR	95% CI	*p-*value	aRR	95% CI	*p-*value
Bed net elevation	0.82	0.72–0.93	0.002	0.75	0.66–0.85	< .001
Quartile 1		REF		–	–	–
No net	–	–	–		REF	
Yes net	–	–	–	0.65	0.53–0.81	< .001
Quartile 2	1.00	0.87–1.16	0.96	–	–	–
No net	–	–	–	0.87	0.70–1.09	0.24
Yes net	–	–	–	0.74	0.61–0.90	0.002
Quartile 3	0.32	0.27–0.39	< 0.001	–	–	–
No net	–	–	–	0.33	0.25–0.44	< .001
Yes net	–	–	–	0.21	0.16–0.28	< .001
Quartile 4	0.20	0.15–0.26	< 0.001	–	–	–
No net	–	–	–	0.15	0.10–0.23	< .001
Yes net	–	–	–	0.15	0.11–0.22	< .001

Unadjusted model regresses RDT result on elevation quartile, and adjusted model regresses RDT result on elevation quartile, bed net use, and their interaction

*CI* confidence interval, *RR* risk ratio, *aRR* adjusted risk ratio

To further explore the relationship between LLIN use and geographic factors, rates of reported LLIN use stratified by distance to the nearest health facility was examined. In the first analysis, the shortest Euclidean (i.e., straight-line) distance to either a level II or level III facility, where LLINs are routinely provided to pregnant women seeking antenatal care and children receiving immunizations, was estimated. Distance from either a level II or III health centre ranged from 0.01 km (11 m) to 6.55 km with a median of 1.12 km and interquartile range 0.70–1.69 km. However, approximately 1 in 7 (15.2%, 95% CI 14.8–15.6) households was located more than 2 km from the nearest health facility. Households at lower elevations were more likely to live closer to healthcare facilities (Table 1). For example, at the lowest three elevation quartiles, approximately half of respondents live less than 1 km from a level II or III health centre, whereas at the highest elevation quartile approximate half of respondents live more than 2 km from a level II or III health centre.

**Fig. 2:**
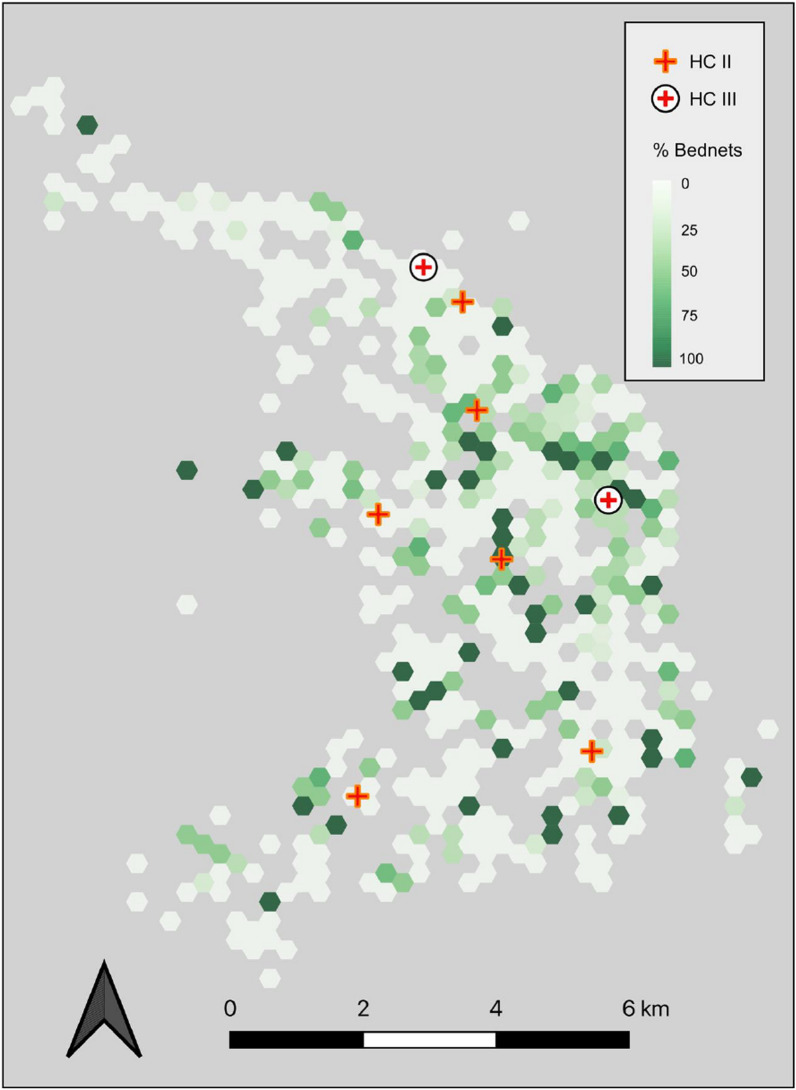
Map displaying the percentage of households that use a LLIN distributed through a health centre compared to all households that use a bed net. Each hexagonal grid represents a minimal diameter of 200 m

As shown in Fig. 2**,** reported LLIN use declined among households living more than 2 km from the nearest level II or level III facility. Compared to those living less than 1 km from a health centre, households at more than 2 km were less likely to report the child sleeping under a LLIN (RR 0.86, 95% CI 0.83–0.89, *p* < 0.001) (Table 3). The analysis was repeated using only the distance to level III facilities, which house the only labour and delivery wards and inpatient units in the sub-county. Again, there was an inverse association between LLIN use and distance to clinic with estimated LLIN use dropping by more than 15% beyond a distance of 4 km.

**Table 3:** Estimated unadjusted (left columns) and adjusted (right columns) risk ratios of LLIN use from log binomial regression modeling

Variable	RR	95% CI	*p-*value	aRR	95% CI	*p-*value
Elevation quartile						
Quartile 1		REF			REF	
Quartile 2	1.06	1.03–1.10	< 0.001	1.07	1.03–1.11	0.02
Quartile 3	1.01	0.97–1.04	0.73	0.97	0.93–1.02	0.23
Quartile 4	0.86	0.83–0.89	< 0.001	0.87	0.81–0.94	< .001
Distance to clinic						
< 1 km		REF			REF	
1–2 km	0.99	0.96–1.01	0.22	1.03	0.98–1.08	0.23
≥ 2 km	0.86	0.83–0.89	< 0.001	0.73	0.64–0.82	< .001

Unadjusted models separately regresses LLIN use on (1) elevation quartile and (2) distance to the nearest level II of level III health centre. Adjusted model regresses LLIN use on elevation quartile and distance to the nearest level II of level III health centre

*CI* confidence interval, *km* kilometre, *RR* risk ratio, *aRR* adjusted risk ratio

**Fig. 3:**
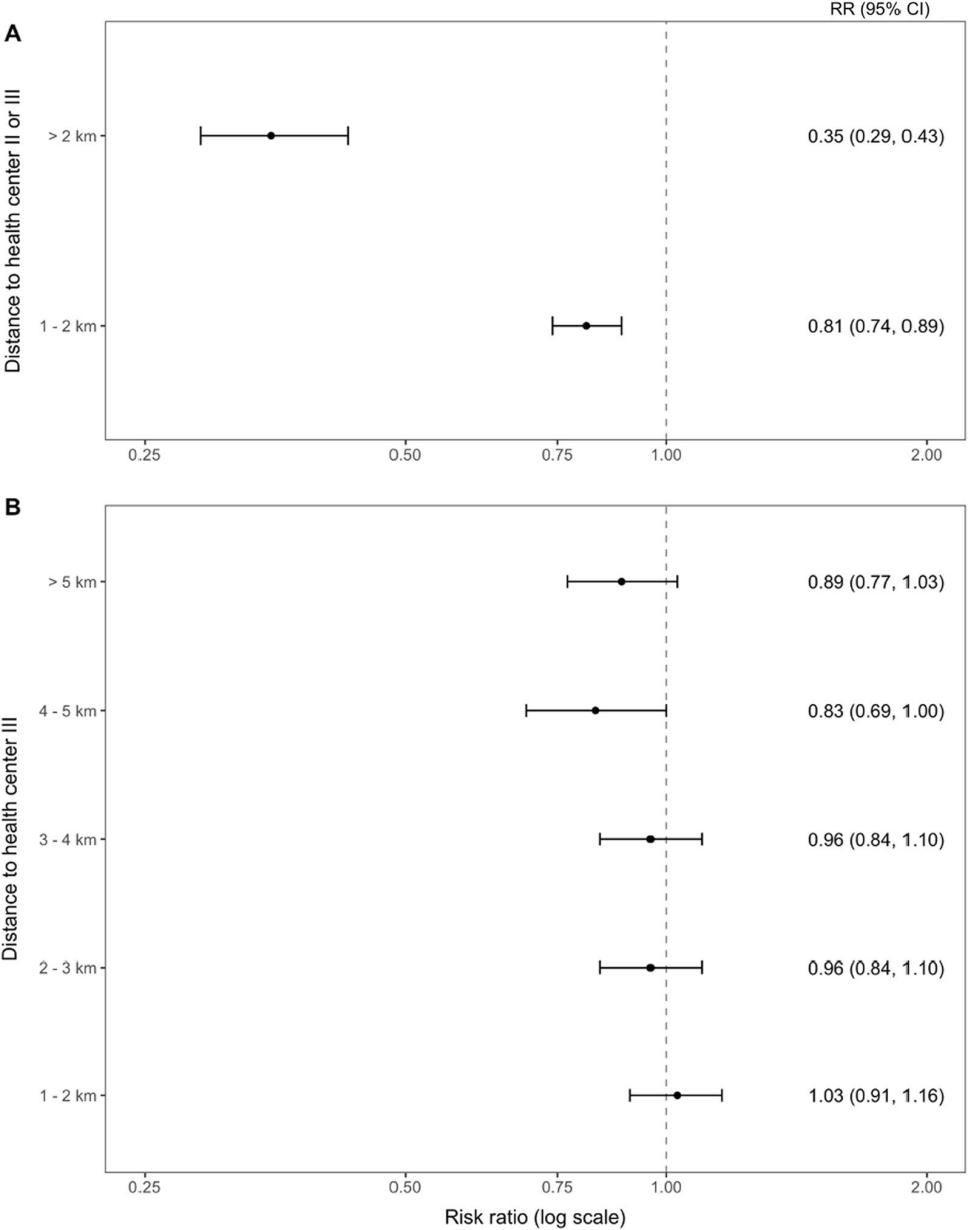
Estimated risk ratios of obtaining LLIN from health centre (versus mass distribution campaign) by distance to health centres. Households living less than 1 km from the health centre are the reference group. Dropped observations where net was reported as “purchased” (n = 4) or “other” (n = 3)

Given that the majority of participants reported obtaining LLINs through a mass distribution campaign, the association between geographic factors and LLIN source was explored. Overall, mass distributions represented the primary source of LLINs across distance categories. However, households located farther from a health centre were more likely to own LLIN sourced from mass distributions, while those located closer to health centres were more likely to own LLINs sourced through clinic visits (Fig. 3). Those who received their LLINs from a mass distribution lived a median distance of 248 m (IQR 184–315 m, *p* < 0.001) farther from a health centre than those who received a LLIN from a health facility. This finding was robust to a sensitivity analysis in which households in the highest elevation quartile, where the majority of surveyed households were located more than 1 km from a health facility, were excluded from the analysis (RR 0.31, 95% CI 0.22–0.44, *p* < 0.001).

## Discussion

Despite Uganda’s substantial commitment to achieving and sustaining universal LLIN coverage and high rates of use, the study—which was conducted near the end of a 3-year government LLIN distribution cycle—found that approximately one-third of surveyed children did not sleep under a LLIN the previous night. The lowest rates of use were observed among households at elevations above 1600 m, where transmission was lower, and those at the greatest distance from health facilities. Given the established correlation between LLIN ownership and use [25], especially among children, our findings suggest that many households did not have adequate access to a LLIN. Improving coverage and use among these most remote households between distribution campaigns remains an important challenge.

The lower LLIN usage rates among those living farther from health facilities likely reflect a greater travel burden and thus may be a surrogate marker of decreased utilization of health facilities for antenatal services and delivery. These are the primary opportunities for households to obtain additional LLINs outside of mass distributions. Notably, households living more than 2 km from health facilities were much more likely to report receiving LLINs from mass distribution campaigns rather than from continuous distributions focused on high-risk patient populations at health facilities. The findings expand upon previous studies showing an inverse association between LLIN ownership or use and distance to a health facility, particularly in rural areas [24, 25]. Given that distance and elevation are relatively easy to obtain from routine sources, these metrics—even if proxy measures—may be valuable as complementary tools to guide implementation strategies, particularly in rural areas.

While the WHO states that “mass campaigns are the only proven cost-effective way to rapidly achieve high and equitable coverage” [8], coverage gaps begin to appear almost immediately post-campaign due to net attrition well before the expiration manufacturer’s 3-year lifespan [33–37]. Previous studies in Uganda have demonstrated the extent to which LLIN coverage and use declines in the interval period between distribution campaigns with only two-thirds of respondents reporting owning at least one LLIN 3 years after the last distribution campaign [13]. While household coverage was not measured, the finding of 65% LLIN use by children (who are often more likely to sleep under LLINs) is consistent with these trends. Furthermore, declines in LLIN coverage and use have been associated with increased parasite prevalence, which highlights the need to develop novel strategies to replace lost and damaged LLINs between distribution campaigns [14].

Continuous distribution through existing health facilities is often cited as an effective supplemental strategy to overcome LLIN attrition [8]. Yet the results shown here suggest that this approach may not lead to high use among children in rural areas, particularly as distance to health facilities increases with the greatest reduction in current use observed beyond a distance of 2 km. Households in these areas appear more dependent on mass distribution campaigns as the primary source of LLINs. In these more remote communities, school-based distributions may sustain higher and more equitable coverage [17, 18, 38]. Uganda also has an established network of CHWs, many of whom already perform evaluation and management of uncomplicated malaria in their homes, who could be leveraged to identify households without adequate LLINs [39–41]. This assessment could take place as a component of febrile illness visits. For example, when a child presents for care, the CHW could ask about LLIN ownership and use as part of the evaluation. CHWs could then distribute additional LLINs if reported coverage is below target levels. Such methods could provide relatively real-time estimates of LLIN coverage at the village level. Community-based malaria case management programmes have been shown to reduce household costs associated with care-seeking and similar benefits might be accrued if LLIN distribution was similarly decentralized [42].

The potential policy implications stemming from the finding of lower reported LLIN use at higher elevations, even when adjusting for distance to clinic, are more nuanced. Above 1400 m, the PfPR declined substantially with a number of the highest elevation villages having no positive RDT results; this result is consistent with the known association between malaria transmission and elevation [43, 44]. Furthermore, there does not appear to be any difference in the risk of malaria parasitemia between children sleeping under a LLIN and those who did not (Table 2). Therefore, residents living at higher elevations may be making conscious decisions not to obtain or not to use LLINs given the lower risk of infection. Low perceived risk has been previously documented as a potential barrier to LLIN use [45]. While staff did not assess travel histories or perform entomologic surveillance [46, 47], it is possible that some, if not most, of the infections identified at higher elevations may have been acquired during travel to lower-elevation market areas or social events (i.e., church, weddings). Given the lower prevalence of infection and minimal expected effect of LLINs on travel-related risk, these findings suggest that, at least from an economic standpoint, LLIN distribution at higher altitudes may be an inefficient use of resources. However, the additional effort and resources required to define discrete altitudinal thresholds at which LLIN distribution campaigns may no longer be effective may not be cost-effective, especially given that most of the Ugandan population resides well below these elevations. Even in low-transmission areas, however, LLIN distribution networks and distribution campaigns may serve other health and non-health goals, such as demonstrating the ability of local government to deliver essential services.

The current study, which was conducted in a setting of highly variable geography and malaria transmission intensity, has a number of strengths including the unique study area and high-proportion of households sampled. There are also important limitations. First, the primary outcomes relied on self-reported outcomes such as LLIN use and source in regard to a single child in each household. Therefore, the study results cannot be extrapolated to estimate household coverage rates. However, it is reassuring that the finding of 64.7% LLIN use is similar to the 68% of children under 5 years of age in the Tooro Region reported to have slept under an LLIN in the most recent MIS, which showed high correlation with LLIN ownership [11]. Furthermore, in a subsequent study conducted in November 2020, notable for occurring shortly after the 2020 mass distribution campaign, rates of LLIN use among children aged 2 to 10 years increased from 65 to 94%, 62% to 92%, and 76% to 94% in the three villages that participated in both surveys. These results highlight the strong relationship between ownership and use and suggest that lack of LLIN remains the main driver of low usage.

Second, participants may also have perceived a social desirability pressure to state that the child had slept under a LLIN. It is reassuring that we observed differences in reported LLIN use across the elevation quartiles, as one would not expect a differential bias by elevation. Third, due to the lack of an established sampling frame (e.g., household addresses), sampling was achieved through a systematic scheme in which staff started at the CHW residence, moved in a clockwise direction, and visited every other household. The non-random start point in each CHW area of responsibility could have introduced bias if ordering of households was associated with malaria risk or LLIN use, but this seems very unlikely, especially given the relatively small geographic area each CHW covers. Lastly, the use of RDTs may not have identified low-density (e.g., < 50 parasites/µL), asymptomatic infections [28]. Given that RDTs are now widely employed for malaria indicator surveys, this seems a reasonable approach and is unlikely to have impacted the conclusions.

## Conclusions

In a setting of variable geography and malaria transmission, LLIN use among children was well below targeted levels. Given that the survey took place approximately 3 years after the last mass distribution campaign and the strong correlation between reported rates of LLIN use and LLIN ownership, these findings suggest that existing continuous distribution efforts may be insufficient. Geographic factors including elevation and distance to health facilities were associated with reported rates of LLIN use and sourcing and may represent accessible measures for targeting supplemental distribution strategies including top-up campaigns, school-based distributions, or novel implementation strategies leveraging existing resources such as CHWs.

## Supplementary Information

**Additional file 1. **K23 Mis Annex 2.

## Data Availability

Deidentified individual data that supports the results will be shared beginning 9 to 36 months following publication provided the investigator who proposes to use the data has approval from an Institutional Review Board, Independent Ethics Committee, or Research Ethics Board, as applicable, and executes a data use/sharing agreement with UNC.

## Abbreviations

BHC: Bugoye Level III Health Centre
C°: Degrees Celsius
CHW: Community Health Worker
CI: Confidence interval
DHS: Demographic and Health Survey
FPC: Finite population correction
HRPII: Histidine-rich protein II
IQR: Interquartile range
LLIN: Long-lasting insecticidal net
Km: Kilometre
M: Meter
MIS: Malaria indicator survey
PfPR: *Plasmodium falciparum* positivity rate
RDT: Rapid diagnostic test
RR: Risk ratio
SSA: Sub-Saharan Africa
