# Supplementary material for: Malaria prevalence and long-lasting insecticidal net use in rural western Uganda: results of a cross-sectional survey conducted in an area of highly variable malaria transmission intensity
Source: Malar J. 2021 Jul 5;20:304. doi: 10.1186/s12936-021-03835-7 (PMC8256478; doi:10.1186/s12936-021-03835-7)
Supplement: Supplementary file 1 — Additional file 1. K23 Mis Annex 2. [file 12936_2021_3835_MOESM1_ESM.pdf]

K23 Mis Annex 2

Study ID

____

Unique Study ID

____
(First 3 letters of village / RA number / Day - Month - Household)

Household Information

Date of visit

____
(DD-MM-YYYY)

Parish

- ◯ Bugoye
- ◯ Ibanda
- ◯ Katooke
- ◯ Kibirizi
- ◯ Muhambo
- ◯ Mubuku

Village

- ◯ Bugoye
- ◯ Kanyanamigho
- ◯ Kisamba 1
- ◯ Kisamba 2
- ◯ Muramba 1
- ◯ Muramba 2
- ◯ Rwakingi 1A
- ◯ Rwakingi 1B

Village

- ◯ Ibanda 1
- ◯ Ibanda 2
- ◯ Nyakabugha
- ◯ Kiharara
- ◯ Mihunga
- ◯ Mirimbo
- ◯ Ruboni

Village

- ◯ Katooke 1
- ◯ Katooke 2
- ◯ Kemihoko
- ◯ Kihindi
- ◯ Kinyangoye
- ◯ Mapata
- ◯ Mulehe
- ◯ Nyangonge
- ◯ Kirongo

Village

- ◯ Bulindiguru
- ◯ Ihani
- ◯ Kasanzi
- ◯ Kibirizi
- ◯ Kikokera

Village	◯ Bunyangoni<br>◯ Katumba<br>◯ Maghoma<br>◯ Muhambo<br>◯ Nduguthu East<br>◯ Nduguthu West
Village	◯ Izinga
Latitude	__________________________________<br>(Automatic Pull)
Longitude	__________________________________<br>(Automatic Pull)
Tablet GPS Working?	◯ Yes<br>◯ No
Latitude (Manual Entry)	__________________________________<br>(Example Format = 0.XXXX)
Longitude (Manual Entry)	__________________________________<br>(Example Format = 30.XXXX)
If your child was sick with a fever, where would you usually seek care?	◯ VHT<br>◯ Drug Shop or Pharmacy<br>◯ Private Clinic or RMS<br>◯ Level II Facility<br>◯ Bugoye Health Center<br>◯ Other
If Level 2 Health Center, which one?	◯ Ibanda HC2<br>◯ Katooke HC2<br>◯ Kibirizi HC2<br>◯ Kisamba HC2<br>◯ Maghoma HC2<br>◯ Nyangonge HC2
If other, please list:	__________________________________

I. Vital Signs - Child

Axillary temperature	__________________________________<br>(degrees Celsius)

II. Medical History - Child

Did the child sleep under a bed net last night?	◯ Yes<br>◯ No
Where did you obtain the net?	◯ Government distribution<br>◯ Health Center<br>◯ Purchase from store<br>◯ Other
Has the child had fever in last two days?	◯ Yes<br>◯ No
If yes, when did the fever start	________________________<br>(DD-MM-YYYY)
In the last 2 weeks, has the child been seen at a hospital, health centre, clinic, drug shop, or other medical attendant for these symptoms?	◯ Yes<br>◯ No
If yes, where?	◯ Hospital<br>◯ Health Centre<br>◯ Drug Shop or Pharmacy<br>◯ VHT<br>◯ Traditional Medicine
Did the child receive medicine for malaria?	◯ Yes<br>◯ No

III. Laboratory Testing - Child

Malaria RDT performed?	◯ Yes<br>◯ No
Malaria RDT Result	◯ Negative<br>◯ Positive

Optional Laboratory Testing - Adult

Does adult choose to have hepatitis B testing?	◯ Yes<br>◯ No
Adult being tested	◯ Mother<br>◯ Father<br>◯ Other caregiver
Hepatitis B RDT Result	◯ Negative<br>◯ Positive
If adult is positive, do they select to test other members of household?	◯ Yes<br>◯ No
If others in the household are tested, please list the age, sex, and RDT result of ALL the others tested.	________________________<br>(Example = (8 - M - POS) or (5 - F - NEG))
